# Obstructive urolithiasis in buffalo calves (*Bubalus bubalis*): Serum changes of Vitamins A and D and efficacy of surgical management using tube cystostomy

**DOI:** 10.14202/vetworld.2021.129-136

**Published:** 2021-01-18

**Authors:** Abdelmonem Abdallah, Shimaa Ezzeldein, Eslam Eisa, Mustafa Abd El Raouf, Yasmin Bayoumi

**Affiliations:** 1 Department of Animal Medicine, Faculty of Veterinary Medicine, Zagazig University, 44519, Zagazig, Sharkia, Egypt; 2 Department of Surgery, Anesthesiology and Radiology, Faculty of Veterinary Medicine, Zagazig University, 44519, Zagazig, Sharkia, Egypt

**Keywords:** buffalo calves, hypovitaminosis, tube cystostomy, ultrasound, urolithiasis

## Abstract

**Background and Aim:**

Obstructive urolithiasis is one of the major health problems in livestock animals, mainly in young calves. The present study was designed first to investigate the changes in the serum levels of Vitamins A and D in buffalo calves (*Bubalus bubalis*) with obstructive urolithiasis and second to investigate the efficacy of tube cystostomy technique in management of such condition.

**Materials and Methods:**

One hundred and forty-nine buffalo calves of variable ages ranged from 3 to 7 months with a history of retained urine were examined clinically and ultrasonographically. Then, they were subjected to surgical treatment using the tube cystostomy technique. The serum levels of Vitamins A and D were investigated in retained urine calves in addition to 10 clinically healthy calves of the same age used as a control group.

**Results:**

Based on clinical and ultrasonographic findings, the calves were diagnosed as obstructive urolithiasis with intact bladder (n=64 calves) or with bladder rupture (n=85 calves) with the peak incidence in winter months. Tube cystostomy was an efficient and quick surgical technique for the management of such condition and 95.3% of calves returned their normal urination within 7–14 days after surgery. Significant hypovitaminoses A and D were found between retained urine calves and control ones (p= 0.01 and 0.002, respectively).

**Conclusion:**

Hypovitaminoses A and D suggested predisposing obstructive urolithiasis in buffalo calves, but further clinical studies are recommended for more confirmation. Surgical treatment using tube cystostomy technique is recommended for the management of obstructive urolithiasis in buffalo calves.

## Introduction

Obstructive urolithiasis is a worldwide disease of major importance in domestic animals. It was found to be the 5^th^ most prevalent cause of death in feedlots [1]. It is described as the concretion of urinary calculi which may lodge anywhere in the urinary system but usually at the distal end of the sigmoid flexure in ruminants [2,3]. Urolithiasis is considered a major problem mainly in males due to the anatomical conformation of their urinary tract [4]. The disease is highly incident in the male buffalo calves than the cow calves. Furthermore, it is more frequently occurred in the winter season rather than other seasons [5-7]. Surgical management is efficient in curing most cases [8]. Surgical interference includes perineal urethrostomy [9], tube cystostomy [4,10-13], urinary bladder marsupialization [14], and penile catheterization and amputation [15].

Urinary calculi formation is a multifactorial process that results from a combination of physiologic, nutritional, and management factors as the diet, age, sex, breed, genetic makeup, season, soil, water, hormone levels, mineral, infection, and other factors usually incorporated in the genesis of urolithiasis [2]. Additional factors have been incriminated as contributing causes, from those factors, the heavy concentrate-low roughage diets, limited intake of water, dehydration, urine alkalinity, mineralized artesian water, alkaline water supplies, excess of sodium bicarbonate in diet, Vitamin A deficiency, and hypervitaminosis D [4,16]. Experimental Vitamin A-free diet was reported to be a risk factor for urinary calculi formation in treated male rats versus a control group [17]. Hypervitaminosis D was previously reported to be a predisposing or risk factor for urinary calculi formation in swine and ruminants [18,19]. In human medicine, the literatures were controversial. A meta-analysis study reported that 25-hydroxyvitamin D serum levels are higher in kidney stone patients [20]. On the other hand, some studies showed a considerable prevalence of Vitamin D deficiency in stone formers compared with non-stone formers [21-24]. Despite the great economic importance of obstructive urolithiasis consequences that could be fatal in some cases, the disease is present and several cases or even outbreaks reported yearly.

The present study aimed first to investigate the changes in the serum levels of Vitamin A and D in buffalo calves (*Bubalus bubalis*) with obstructive urolithiasis and second to investigate the efficacy of tube cystostomy technique in management of such condition.

## Materials and Methods

### Ethical approval

The study protocol was designed according to the Ethics Committee of the Egyptian Veterinary Medicine Authority.

### Study location, period and Animals

A total of 149 non-castrated male buffalo calves aging 3-7 months were admitted to the clinic of the Faculty of Veterinary Medicine, Zagazig University, with a history of retained urine during the period between June 2018 and June 2020. Ten apparently healthy non-castrated male buffalo calves of the same age were used as a control group for biochemical analysis.

### Clinical examination

All calves were undergone a thorough clinical examination as described previously [25]. Data concerning age, days of retention, and food and water intake were recorded. Rectal body temperature, respiration rate, and heart rate were investigated using standard techniques. The color of the eye mucus membrane and the dehydration degree through skin tent test were examined. Abdominal distension and ballottement for fluid thrilling were reported. Abdominocentesis at the left paramedian site behind umbilicus was performed using a sterile 18-gauge needle under aseptic precautions in standing animals. Preoperatively, severely dehydrated animals were stabilized with fluid therapy as required and then operated with tube cystostomy on the same day.

### Biochemical analysis

Before surgical interference, blood samples were drained from the jugular vein of the affected calves in plain sterile tubes without anticoagulant, then centrifuged and sera were harvested. Spectrophotometrically, serum calcium, inorganic phosphorus, creatinine, and blood urea nitrogen levels were determined by standard procedures using (Diagnostic Zrt. Commercial kits, Biomerieux). Serum levels of Vitamins A and D were measured using ELISA kits (Cat No. MBS267174 and CSB-E0809r, respectively).

### Ultrasonographic examination

All retained urine calves were examined for the urinary bladder condition using 5 MHz transabdominal transducer (Sonoscape A5V, China) in standing position just cranial to pelvic rim. Furthermore, a 6 MHz transrectal transducer was used to examine the pelvic urethra.

### Surgical management

**Figure-1:**
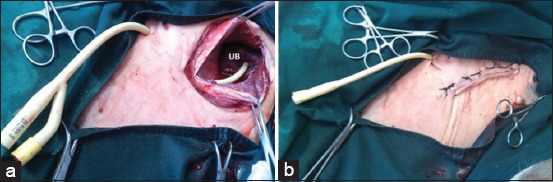
Tube cystostomy technique. (a) Foley catheter implantation inside the bladder through an abdominal incision. (b) Fixation of the Foley catheter outside the body and suturing the abdominal incision. UB=Urinary bladder.

All retained urine calves were subjected to tube cystostomy technique as described previously [3,4,11-13,26]. Before surgery, the calves were secured in dorsal recumbency. The area from the pubic bone until umbilicus was aseptically prepared. Linear infiltration anesthesia was performed using lidocaine HCL 2% (Debocaine^®^, the Arab Company, Obour City, Egypt) at the left paramedian region extended 10 cm cranially from the rudimentary teat. Calves with bladder rupture were sedated using 0.2 mg/kg of Xylazine HCL 2% (Xyla-Ject^®^, ADWIA Pharmaceuticals Co., 10^th^ of Ramadan City, Egypt). A 10 cm skin and S/C incision were performed at the anesthetic site parallel to preputial sheath, then the muscle and peritoneum were incised. Through a new 1 cm abdominal wall incision cranial to the first one, 18 French Foley catheter was implanted in the abdomen, then inside the bladder lumen through a stab incision in its wall (Figure-1). The catheter was fixed in the bladder and abdominal wall by simple suture. In case of the bladder rupture, cystorrhaphy was performed with Cushing suture pattern using Vicryl No. 1 sutures followed by Foley catheter placement after necessary debridement of necrotic parts and removal of concretion and cystic calculi through irrigation with normal saline solution. Peritoneal lavage was performed using sterile 0.9% saline solution. The abdominal incision was then routinely closed, and the skin was closed with horizontal mattress sutures using silk No. 2.

Parenteral administration of antibiotic (Pen&Strep^®^, Norbrook Co., N. Ireland, 1 mL/25 kg) for successive 5 days and anti-inflammatory (flunixin meglumine, Flunixin^®^, Norbrook Co., N. Ireland, 2.2 mg/kg) for successive 3 days was performed. The operated calves were orally administered 10 g of ammonium chloride dissolved in 40 mL of water for 20 days postoperatively. Intermittent blockade of the Foley catheter for 30 min 3 times daily until normal urination has occurred. Skin stitches were removed within 12 days after surgery.

### Statistical analysis

The obtained data were analyzed using R3.1.3 software (R Foundation for Statistical Computing, Vienna, Austria). The Mann–Whitney U-test was used to investigate the statistical difference between serum levels of Vitamins A and D, Ca, Ph, urea, and creatinine in control and diseased calves as their values were not normally distributed. Statistical significance was set at p< 0.05. The percentage of urine retention cases per month during the study period (2018-2020) calculated using the following formula: Retained urine cases % per month=n (sum of retained urine cases per month)/total number of cases during the study period. The Kruskal–Wallis test was used to assess the seasonal variation of cases.

## Results

Based on clinical and ultrasonographic findings, the calves were diagnosed as obstructive urolithiasis with intact bladder (n=64 calves) or with bladder rupture (n=85 calves).

### Clinical findings

The clinical findings of calves with retained urine are summarized in Table-1. Calves with intact bladder showed signs of anuria, anorexia, abdominal pain, urethral pulsation, and raised tail with protrusion of anal mucous membrane. On the other hand, calves with bladder rupture showed signs of anuria, pear-shaped abdomen with fluid thrilling in 85.9% of calves, decreased food intake, sunken eye in 87.1% of calves, dehydration, uremic breath in 62.3% of calves, and roughened coat with bran-like scales. Recumbency was reported in 16.5% of calves with bladder rupture. The duration of retention ranged from 1 to 3 days in calves with intact bladder and from 4 to 7 days in calves with bladder rupture. Abdominocentesis revealed presence of free urine inside the abdomen in calves with bladder rupture.

**Table-1:** Clinical findings in buffalo calves with obstructive urolithiasis.

Parameters	Calves with obstructive urolithiasis (n=149)			

	Intact bladder (n=64)		Bladder rupture (n=85)	
	
	n	%	n	%
Restlessness	64	100	8	9.4
Dullness and depression	0	0	76	89.4
Anorexia	47	73.4	65	76.5
Sunken eyes	19	29.7	74	87.1
Congested conjunctival mucosa	37	57.8	66	77.6
Alteration in vital signs	29	49.1	62	72.9
Reduced or absent Rumen motility	37	57.8	65	76.5
Abdominal pain syndrome	57	89.1	0	0
Pear-shaped distended abdomen	0	0	73	85.9
Detectable fluid thrill abdomen	0	0	80	94.1
Difficulty in movement	0	0	22	25.9
Roughness of the coat	34	53.1	73	85.9
Bran-like scale on the skin	30	46.9	50	58.8
Uremic breath	0	0	53	62.3
Urethral pulsation	51	79.7	0	0
Rectal prolapse	3	4.7	2	2.3
Recumbency	0	0	14	16.5

The percentages of retained urine in buffalo calves within different seasons during the study period are summarized in Table-2. Non-significant differences between the percentages of cases based on seasons (p=0.44) were observed with peak incidence during the winter months with respective percentages of 17.5%, 21.5%, and 19% for December, January, and February.

**Table-2:** Monthly distribution of retained urine cases.

Months	Cases	Percentage
January	32	21.5
February	28	19
March	16	10.5
April	8	5.5
May	1	0.5
June	1	0.5
July	1	0.5
August	1	0.5
September	2	1.5
October	11	7.5
November	22	15
December	26	17.5

### Biochemical findings

The biochemical findings of the retained urine calves and normal calves are illustrated in Table-3. The serum levels of the Vitamins A and D in retained urine calves were significantly lower than those of the control ones (p=0.01 and 0.002, respectively). Furthermore, the serum levels of calcium in retained urine calves were significantly lower than the control calves (p=0.03), while the serum levels of urea and creatinine in calves suffered urine retention were significantly higher than the control ones (p=0.01 and 0.02, respectively).

**Table-3:** Serum parameters in healthy and retained urine calves reported as mean±SD and ranges.

Serum parameters	Healthy group			Urine retention group		
	
	Mean±SD	Min	Max	Mean±SD	Min	Max
Vitamin A (µg/dL)	43.2±10.3	31.9	52.2	7.3±4.4	2.4	13.7
Vitamin D (ng/mL)	39.4±12.9	24.9	49.8	8.3±4.9	1.1	14.8
Urea (mg/dL)	43.8±13.5	31.3	58.2	295±80	227.1	432.4
Creatinine (mg/dL)	1.1±0.3	0.8	1.5	8.65±7.8	0.9	25.1
Calcium (mg/dL)	9.8±1	8.7	10.6	6.4±2.5	1.4	9.8
Phosphorus (mg/dL)	7.2±0.3	6.8	7.5	8.7±0.9	6.7	9.7

SD=Standard deviation

### Ultrasonographic findings

**Figure-2:**
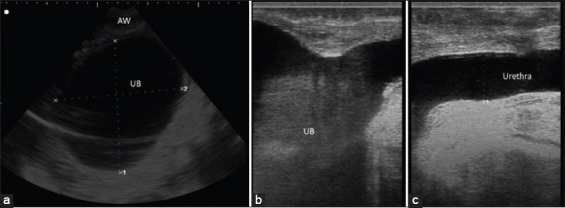
Ultrasonographic scanning of bladder and urethra. (a) Transabdominal ultrasonographic image of distended bladder in a male buffalo calf at the ventral abdomen in front of the scrotum after 2 days from retention. The bladder appeared as a distended anechoic sac with a hyperechoic wall. (b and c) Transrectal ultrasonographic image of the distended bladder and urethra, respectively, in the male buffalo calf. Note the distended pelvic urethra. UB=Urinary bladder; AW=Abdominal wall.

**Figure-3:**
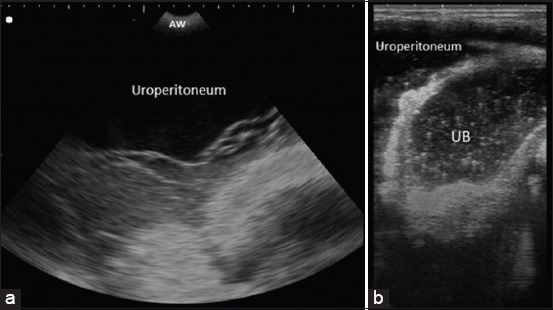
Ultrasonographic scanning of bladder. (a) Transabdominal ultrasonographic image of the bladder rupture in a male buffalo calf at the ventral abdomen in front of the scrotum after 5 days from retention. Note free anechoic fluid inside the abdomen. (b) Transrectal ultrasonographic image of the bladder rupture in the male buffalo calf. The bladder appeared smaller than normal with a thickened hyperechoic wall with anechoic fluid infiltration in the abdomen (uroperitoneum). UB=Urinary bladder; AW=Abdominal wall.

The ultrasonographic imaging of calves with intact bladder showed distended anechoic bladder sac with circumscribed hyperechogenic contour inside the abdomen in transabdominal examination and distended pelvic urethra to be more than 1 cm when examined transrectally (Figure-2). While, the sonographic picture of calves with ruptured bladder showed free anechoic fluid inside the abdomen and the bladder appeared smaller in size (Figure-3).

### Surgical outcomes

During the surgical procedure, the urinary bladder was found intact in 64 calves and ruptured in 85 calves. The site of bladder rupture was varied from dorsum to ventral aspect and vertex to neck of the bladder. The dorsal neck rupture was reported in 61 cases while dorsal vertex was reported in 11 cases. The ventral vertex was reported in five cases, while subserous rupture was reported in nine cases. The bladder appearance was smooth, and its color varied from pinkish to dark blue in 64 calves with intact bladder. In 61 calves with bladder rupture, the urinary bladder was rough, corrugated, and necrosed. Furthermore, the bladder was adhered to the peritoneum, omentum, mesentery, or intestine in 15 calves with bladder rupture.

All operated calves were followed up until normal urination has occurred. About 95.3% of calves (n=145) returned normal urination within 7-14 days after surgery, while 2% of calves returned normal urination after 30 days from surgery. Only 2.7% of the operated calves showed complications of Foley catheter loosening and obstruction. The Foley catheter was removed after resuming normal urination from the external urethral orifice and the owners were advised to supply more water and less concentrated feeding. About 128 calves were followed for 5 months after surgery without recurrence.

## Discussion

Urolithiasis is a life-threatening disease of ruminants and of high economic importance to the farmers due to a high mortality rate resulted from the ruptured urinary bladder and the treatment costs [4]. Recent Egyptian studies were conducted regarding this condition [7,13], the former study reported the comparative diagnosis of retained urine cases, while the latter one discussed the clinical findings, serum analysis, and the surgical management of obstructive urolithiasis in male buffalo calves. These studies lack the evaluation of serum levels of Vitamins A and D.

The age of all admitted calves was ranged from 3 to 7 months. This age was susceptible for obstructive urolithiasis in buffalo calves as reported in the previous studies [4,7,13]. Such findings might be attributed to the weaning of male buffalo calves that accompanied by diet shifting to high concentrates, and this could be favored by limited water intake in cold months as presented in the present study [27].

Nearly 58% of retained urine cases were recorded in winter months (December, January, and February) throughout the study period, although the seasonal variation was non-significant. These findings were consistent with the previous studies [2,6,27,28]. On the other hand, a previous study reported a higher incidence of urolithiasis in summer [29]. Decreased water intake with high concentrate feeding and low carotene-containing berseem that usually fed in winter could account for higher cases of incidence during winter months [27].

The most reported signs related to urolithiasis with intact bladder were anuria, restlessness, anorexia, abdominal colic pain, urethral pulsation, and raised tail. These signs agreed with previously described [30]. Contrastingly, calves with bladder rupture showed signs of anuria, anorexia, dullness, dehydration, sunken eye, abdominal distension with thrilling, rough coat, and uremic breath, these findings were reported in calves with bladder rupture in the previous studies [7,11].

Alterations in the vital signs were reported in all calves suffered urolithiasis with intact or ruptured bladder. The changes in vital signs and general condition of the diseased calves were correlated to the duration of retention and the condition of the urinary bladder. In general, the calves with short duration of retention and kept bladder intact were in superior condition than calves with long duration of retention and bladder rupture with accumulation of urine in the peritoneal cavity (uroperitoneum). This might be related to the metabolic changes associated with uroperitoneum [29].

In the present study, the duration of retention may give a tentative diagnosis of the case, anuria for 1-3 days indicating intact bladder while anuria for more than 3 days indicating complications with bladder rupture. Similar observations were reported previously [7,29]. The long duration of the illness might be attributed to lack of attention by the owners or delay of admission due to misdiagnosis of the cases and failed treatment trials [7]. Ultrasonography is a good diagnostic tool for different internal disorders, especially in cattle and buffaloes [31,32]. It is helpful in determining the conditions of the urinary system, especially the kidneys and urinary bladder [7,33,34]. It is considered the primary diagnostic imaging technique in diagnosis of cases with obstructive urolithiasis [35]. In the present study, ultrasonography was very helpful and diagnostic in cases with bladder atony. It helps to visualize the bladder contour and uroperitoneum in bladder rupture cases [7,34].

In normal healthy calves, the urinary bladder appeared round to oval anechoic sac inside the pelvis in transabdominal examination [36]. While in calves with intact bladder, the urinary bladder appeared severely distended sac with distal acoustic enhancement inside the abdomen in transabdominal examination. In transrectal examination, the pelvic urethra appeared distended with increased diameter of more than 1 cm. On the other hand, the transabdominal examination of calves with bladder ruptures revealed small-sized bladder sac with thickened hyperechogenic wall and floating omentum and viscera in free anechoic fluid inside the abdomen. Similar imaging features were observed in the previous studies [37].

The serum levels of urea and creatinine in the retained urine calves were significantly higher than the control ones. These changes might be due to reduce the rate of glomerular filtration due to the back pressure on the kidneys in intact bladder cases and increase absorption of urea and creatinine from the peritoneal cavity in bladder rupture cases [38]. It was reported that the measurement of serum urea and creatinine is considered the first step in diagnosing the defects in metabolic wastes excretion from the body [7]. The serum levels of phosphorus were higher in the retained urine calves than the healthy ones. Furthermore, secondary hypocalcemia to higher phosphorus level was reported in retained urine calves due to higher excretion of calcium in the urine. Same findings were reported in a previous study [7].

Vitamin A is very important vitamin and is required for healthy epithelization of the tissues. In addition, it has an important role in balance of the Vitamin D concentration [2]. Therefore, desquamation of urinary epithelium and insoluble urinary minerals and salts may be a suitable nidus for the formation of urinary calculi [18]. In the present study, hypovitaminosis A was significantly in the retained urine calves in comparison to the control ones. From this point, lowering the serum levels of Vitamin A may be considered a potential factor for occurrence of urolithiasis.

In this study, a significant hypovitaminosis D was found in retained urine cases compared to the control ones. Our findings agreed with some human medical studies [21-24] that found lowered Vitamin D levels in stone former patients. Vitamin D deficiency induces oxidative stress and kidney inflammation that might be a possible risk factor for stone formation [39]. On the other hand, hypervitaminosis D was reported to be associated with kidney stones formation that might be attributed to increase urinary calcium levels [18,19,40].

Intraoperatively, the urinary bladder was found intact in 64 calves and ruptured in 85 calves. The site of bladder rupture was varied from complete dorsal neck (n=61 calves) to dorsal vertex (n=11 calves) and ventral vertex (n=5 calves). The incomplete subserous rupture was reported in nine calves. The reason of bladder rupture may be due to weakness of the bladder muscles and transitional epithelium in the dorsum. The previous studies observed the rupture most commonly at the dorsum [41]. The subserous rupture may be due to the separation of the muscles rather than tearing. Smooth bladder surface with pinkish to dark blue color in 64 calves having intact bladder might be due to bladder over-stretching resulting venous congestion. Rough, corrugated, and necrosed bladder surface reported in 61 calves having bladder rupture might be due to contraction of the bladder after rupture. Adhesion of the bladder to the peritoneum, omentum, mesentery, or intestine in 15 calves might be due to their attachment with rough surface of the bladder after rupture.

The treatment of obstructive urolithiasis in ruminants is primarily surgical [42]. Tube cystostomy technique was found to be successful, rapid, simple, and easily applied procedure for treatment [3,4,11-13,26,43,44]. It has advantages over other surgical techniques of preservation breeding function of the animal and urinary continence [14]. All calves with intact bladder were treated surgically with tube cystostomy while calves with bladder rupture were treated with cystorrhaphy followed by tube cystostomy. Interestingly, most of the treated calves with tube cystostomy (95.3%) had their normal flow of urination within 7-14 days postoperatively. These findings were in accordance with the previous studies [3,13,26,14]. This early response might be attributed to many factors; (1) reduction in urethral inflammation and spasm through administration of anti-inflammatory, (2) drying of the calculi after bladder evacuation using Foley catheter, (3) dissolving of urine using acidic agent such as ammonium chloride orally, and (4) in addition to bladder flushing using sterile saline solution [44,45]. Daily oral administration of 10 g ammonium chloride as urinary acidifier along with tube cystostomy has a synergistic effect for quick recovery of animals as suggested previously [3,41,43].

In the present study, recurrence of the condition was not noticed in successfully treated calves during post-operative follow-up period of 5 months. It has been considered that the success of the surgical procedure when recurrence of the obstruction did not recur [46].

The surgical procedure was performed under local infiltration anesthesia at the site of surgery with satisfactory results. A similar anesthetic protocol was performed previously for surgical treatment of obstructive urolithiasis in ruminants using tube cystostomy technique [3,47,48].

The complication of tube cystotomy in the present study was reported in 2.7% of the cases due to catheter loosening may be due to deflation of catheter bulb and catheter obstruction with blood or tissue debris. Prevention of recurrent obstructive urolithiasis was addressed by the use of urinary acidifiers and dietary management.

## Conclusion

Obstructive urolithiasis was found to be highly incident in male buffalo calves. Young ages between 3 and 7 months, winter season, and hypovitaminoses A and D could be potential risk factors for occurrence. Tube cystostomy using Foley catheter was a practicable, quick, and reliable method for the management of such condition. Future large cohort studies are recommended to confirm the role of hypovitaminoses A and D in urolithiasis. The owners should be impressed with the importance of water consumption for animals with the risk of urolithiasis. This could be achieved by addition of salts to the ration to promote intake of large amounts of water and formation of large volume of diluted urine. Supplementation of vitamins to the ration on months of high incidence is very important in the prevention of urolith formation.

## Authors’ Contributions

All authors designed, planned, drafted, and revised the manuscript. AA contributed to statistical analysis. SE, EE, and MA contributed to clinical and ultrasonographic examinations in addition to surgical interference and post-operative follow-up of cases. YB contributed to clinical and ultrasonographic examinations. All authors read and approved the final manuscript.
